# Proof of surgical publication prowess: prospective observational study of factors associated with surrogate markers of academic reach

**DOI:** 10.1002/bjs5.50307

**Published:** 2020-06-03

**Authors:** D. B. T. Robinson, A. G. M. T. Powell, L. Hopkins, O. P. James, T. Abdelrahman, R. Egan, W. G. Lewis

**Affiliations:** ^1^ Health Education and Improvement Wales School of Surgery Nantgarw UK; ^2^ Division of Cancer and Genetics Cardiff University Cardiff UK; ^3^ Department of Surgery, Morriston Hospital Swansea UK

## Abstract

**Background:**

In the UK, general surgery higher surgical trainees (HSTs) must publish at least three peer‐reviewed scientific articles (as first, second or corresponding author) to qualify for certification of completion of training (CCT). This study aimed to identify the factors associated with success in this arena.

**Methods:**

Deanery rosters supplemented with data from the Intercollegiate Surgical Curriculum Programme, PubMed and ResearchGate were used to identify the profiles of consecutive HSTs. Primary outcomes were publication numbers at defined points in higher training (speciality training year (ST) 3–8); secondary outcomes were the Hirsch index and ResearchGate scores.

**Results:**

Fifty‐nine consecutive HSTs (24 women, 35 men) were studied. The median publication number was 3 (range 0–30). At least three published articles were obtained by 30 HSTs (51 per cent), with 19 (38 per cent) of 50 HSTs achieving this by ST4 (of whom 15 (79 per cent) had undertaken out of programme for research (OOPR) time) and 24 (80 per cent) by ST6. Thirteen HSTs (22 per cent) (ST3, 6; ST4, 4; ST5, 2; ST8, 1) had yet to publish at the time of writing. OOPR was associated with achieving three publications (24 of 35 (69 per cent) *versus* 6 of 24 (25 per cent) with no formal research time; *P* = 0·001), higher overall number of publications (median 6 *versus* 1 respectively; *P* < 0·001), higher ResearchGate score (median 23·37 *versus* 5·27; *P* < 0·001) and higher Hirsch index (median 3 *versus* 1; *P* < 0·001). In multivariable analysis, training grade (odds ratio (OR) 1·89, 95 per cent c.i. 0·01 to 3·52; *P* = 0·045) and OOPR (OR 6·55, 2·04 to 21·04; *P* = 0·002) were associated with achieving three publications.

**Conclusion:**

If CCT credentials are to include publication profiles, HST programmes should incorporate research training in workforce planning.

## Graphical abstract

This study aimed to identify the factors associated with success in this arena. Out of programme for research time was independently associated with attaining certification of completion of training (CCT) publication requirements (odds ratio 6·55, 95 per cent c.i. 2·04 to 21·04; *P* = 0·002). If CCT credentials are to include publication profiles, higher surgical trainee programmes should incorporate research training in workforce planning.

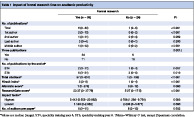

Research training required

## Abstract (es)

**Antecedentes:**

En el Reino Unido, para obtener el título de especialista (*certification of completion of training*, CCT), los residentes de cirugía general durante la etapa de formación específica (*higher general surgical trainees*, HST) deben publicar, al menos, tres artículos científicos en revistas con sistema de revisión por pares (*peer review*) (como primer o segundo autor o como autor para la correspondencia). Este estudio tuvo como objetivo identificar los factores asociados con el éxito en este aspecto.

**Métodos:**

Se identificaron las reseñas de HST consecutivos, mediante datos propios de cada institución y del Intercollegiate Surgical Curriculum Programme, PubMed y ResearchGate. La variable principal fue el número de publicaciones en puntos definidos de la etapa de formación específica (ST3‐8); las variables secundarias fueron los índices de Hirsch y las puntuaciones de ResearchGate.

**Resultados:**

Se analizó la actividad científica de 59 HST consecutivos (24 mujeres, 35 varones). La mediana del número de publicaciones fue de 3 (rango 0‐33). Treinta HST (50,8%) lograron >3 publicaciones; 19 (38,0%) lo lograron en ST4 (78,9% durante el período de investigación al margen del programa de formación quirúrgica (*Out of Programme Research* (OOPR)), y 24 (80,0% de la totalidad de la cohorte) en ST6. Trece HST (22,0%) no habían publicado ningún trabajo hasta el momento de la redacción de este artículo (6 ST3, 4 ST4, 2 ST5 y 1 ST8). El OOPR se asoció con la consecución de las 3 publicaciones (68,6% *versus* 25,0%, *P* = 0,001), con un mayor número de publicaciones (mediana 6 *versus* 1, *P* < 0,001), con puntuaciones ResearchGate más elevadas (23,37 *versus* 5,27, *P* < 0,001) e índices de Hirsch más altos (3 *versus* 1, *P* < 0,001). En el análisis multivariable, el año de residencia (razón de oportunidades, *odds ratio*, OR 1,890, i.c. del 95% 0,014‐3,522, *P* = 0,045) y el OOPR (OR 6,545, i.c. del 95% 2,037‐21,036, *P* = 0,020) se asociaron con la consecución de las tres publicaciones.

**Conclusión:**

Si la CCT exige un número de publicaciones, los programas de los HST deberían incorporar formación en investigación dentro de la actividad laboral habitual.

## Introduction

Surgical scientific journal publication now plays a role that was not part of the original job description, as a metric of surgical trainees' academic prowess and therefore career progression. However, the level of proof demanded by UK Joint Committee of Surgical Training (JCST) Speciality Advisory Committees regarding such academic reach and publication veracity varies widely.

Minimum academic requirements set by the JCST outline the absolute minimum number of publications, communications to learned societies and audits required for certification of completion of training (CCT)^1^, ^2^. For certain specialties (oral and maxillofacial surgery (OMFS), plastic surgery, trauma and orthopaedic surgery), publications and/or presentations may be included as part of the minimum requirements, but could technically be substituted with predetermined equivalents, including higher degrees and patient recruitment into research projects^2^. Publication requirements range from zero (OMFS, trauma and orthopaedic surgery) to four (cardiothoracic surgery, paediatric surgery). Similarly, national presentation requirements range from zero in six of ten specialties to six in cardiothoracic surgery. Audit requirements are also variable, ranging from one to six per training programme (median 4). UK general surgery higher surgical trainees (HSTs) are required to publish at least three peer‐reviewed scientific articles, with the additional caveat that only first, second or corresponding author status qualifies for CCT.

In stark contrast, in the arena of legal training, no such academic competencies exist in relation to published works^3^. In this equally esteemed arena, competence is judged by the completion of either a qualifying law degree or a degree in any other subject, supplemented by the Common Professional Examination (CPE), or an approved Graduate Diploma in Law course; no stipulations are made regarding legal peer‐reviewed publications. This raises the spectre of philosophical relativism^4^, an idea that views are relative to different perceptions and considerations; that there are no universal objective truths, but rather each point of view has its own truth. This study aimed to identify factors associated with success at achieving peer‐reviewed scientific publication success within the setting of a single UK deanery.

## Methods

Consecutive HSTs within the Wales Deanery were identified through deanery records and Intercollegiate Surgical Curriculum Programme (ISCP) profiles using Head of School function. Publication details for each trainee, Hirsch (h) indices, number of citations and impact factor were obtained from PubMed in concordance with ISCP self‐completed evidence entries. Altmetric it! bookmarklet (https://www.altmetric.com/products/ free‐tools/bookmarklet) was used to obtain Altmetric scores for all publications. This methodology has been described previously^5^, ^6^, ^7^, ^8^.

Data were also collected on whether trainees had a ResearchGate profile and their associated ResearchGate score. Primary outcome measures were publication numbers at defined points in higher training (specialty training year (ST) 3–8); secondary outcomes were h‐indices and ResearchGate scores.

### Exclusion and inclusion criteria

Exclusion criteria were case reports or technique reports, collaborative studies, and any non‐medical publications from degrees or research undertaken before entry into medical school. Inclusion criteria were any publication since the commencement of medical school that did not meet any of the exclusion criteria, in order to be in line with the current CCT guidelines set by the JCST^2^.

### Statistical analysis

All data were expressed as median (range), and non‐parametric inferential statistical methods were used throughout. Univariable and multivariable logistic regression models were developed to identify independent associations with the primary outcome measures. Univariable analyses were performed using Mann–Whitney *U* and Kruskal–Wallis tests. Spearman's correlation coefficient was used to test the relationships between paired data sets. Binary logistic regression using a forward conditional model was used to perform the multivariable analysis. All statistical analysis was performed using SPSS® version 25.0 (IBM, Armonk, New York, USA).

## Results

Data were available for 59 consecutive HSTs (24 women, 35 men). The overall median number of publications was 3 (range 0–30), with 30 (51 per cent) achieving a minimum of three publications. Thirteen trainees (ST3, 6; ST4, 4; ST5, 2; ST8, 1) had yet to publish a single paper at the time of writing. Of the 50 trainees who had reached the end of ST4, 19 (38 per cent) managed to achieve three publications by this time, of whom 15 (79 per cent) had undertaken out of programme for research (OOPR). Of the 30 specialty registrars (StRs) who had completed their ST6 training year, 24 (80 per cent) had achieved three publications.

### Impact of formal out of programme for research time

Formal OOPR was undertaken by 35 (59 per cent) of the 59 trainees, of whom 24 (69 per cent) had completed their research, with the others remaining in OOPR. Of these 35 StRs, 22 (63 per cent) had undertaken this research as HSTs, with 12 (34 per cent) undertaking this experience before higher surgical training, and one (3 per cent) both before and during higher surgical training. Undertaking OOPR before higher surgical training was associated with a greater likelihood of success in attaining three publications by the end of ST4, compared with OOPR undertaken as an HST (10 of 13 (77 per cent) *versus* 6 of 22 (27 per cent) respectively; median number of publications 5 *versus* 1, *P* = 0·002), but this advantage was lost by completion of the ST6 training year (10 of 11 (91 per cent) *versus* 11 of 13 (85 per cent); median number of publications 6 *versus* 7, *P* = 0·366).

*Table* *1* highlights the impact of undertaking OOPR on academic productivity and bibliometric analysis. Of the 11 individuals who had not achieved three publications, despite having OOPR experience, nine (82 per cent) were still engaged in research at the time of writing. Only two (8 per cent) of 24 StRs who had completed formal OOPR had not successfully published three articles.

**Table 1:** Impact of formal research time on academic productivity

	Formal research		
	Yes (*n* = 35)	No (*n* = 24)	*P* †
**No. of publications** *			
Total	6 (0–30)	1 (0–6)	< 0·001
1st author	2 (0–10)	0 (0–2)	< 0·001
2nd author	1 (0–11)	0 (0–4)	0·002
Last author	0 (0–4)	0 (0–1)	0·200
Middle author	1 (0–12)	0 (0–2)	< 0·001
**Three publications**			0·001‡
Yes	24	6	
No	11	18	
**No. of publications by the end of** *			
ST4	2 (0–11)	1 (0–4)	0·037
ST6	6 (0–21)	3 (0–6)	0·019
**Total citations** *	57 (0–657)	1 (0–106)	< 0·001
**Hirsch index** *	3 (0–9)	1 (0–4)	< 0·001
**Altmetric score** *	1 (0–278)	0 (0–12)	0·060
**ResearchGate score** *	23·37 (0–37·79)	5·27 (0–17·5)	< 0·001
**Impact factor** *			
Highest	5·443 (0·509–23·562)	2·766 (1·268–6·754)	0·003
Lowest	1·145 (0‐2·962)	2·096 (0–3·877)	0·060
**No. of authors per paper** *	6 (3–11)	6 (2–13)	0·552

* Values are median (range). ST4, speciality training year 4; ST6, speciality training year 6.

† Mann–Whitney *U* test, except

‡ Spearman correlation.

### Bibliometric analysis based on sex

Despite a significantly smaller proportion of the women in this cohort undertaking OOPR (10 of 24 (42 per cent) *versus* 25 (71 per cent) of the 35 men; *P* = 0·022), there were no significant variations in their academic output compared with that in men (*Table* *2*).

**Table 2:** Bibliometric analysis based on sex

	Men (*n* = 35)	Women (*n* = 24)	*P* †
**Formal research**			0·022‡
Yes	25	10	
No	10	14	
**No. of publications** *			
Total	4 (0–30)	2 (0–17)	0·379
1st author	1 (0–9)	1 (0–10)	0·166
2nd author	1 (0–11)	1 (0–6)	0·738
Last author	0 (0–2)	0 (0–4)	0·735
Middle author	1 (0–12)	1 (0–8)	0·245
**Three publications**			0·532‡
Yes	19	11	
No	16	13	
**No. of publications by the end of** *			
ST4	2 (0–11)	1 (0–11)	0·091
ST6	6 (0–21)	5 (1–12)	0·800
**Total citations** *	8 (0–657)	5 (0–129)	0·236
**Hirsch index** *	2 (0–9)	1 (0–6)	0·354
**Altmetric score** *	1 (0–278)	0 (0–244)	0·114
**ResearchGate score** *	22·26 (0–37·79)	16·2 (0–36·41)	0·157
**Impact factor** *			
Highest	5·899 (0·509–23·562)	3·294 (1·268–7·280)	0·005
Lowest	1·145 (0–3·688)	1·760 (0–3·877)	0·366
**No. of authors per paper** *	6 (3–13)	6 (2–11)	0·434

* Values are median (range). ST4, speciality training year 4; ST6, speciality training year 6.

† Mann–Whitney *U* test, except

‡ Spearman correlation.

### Specialty interest and laboratory‐based *versus* clinical research variations

Bibliometric variations based on specialty interest of research undertaken and variations in productivity between individuals carrying out laboratory‐based *versus* clinical research are outlined in *Table* *3*.

**Table 3:** Out of programme research productivity by specialty interest and laboratory *versus* clinical research

		No. of publications by		
	No. of trainees	ST4	ST6	Overall no. of publications
**Specialty**				
UGI	5	3 (0–7)	6 (3–21)	7 (4–21)
Colorectal	8	4·5 (0–11)	7 (6–19)	8 (1–30)
Transplant	3	2 (0–2)	6*	2 (0–11)
Education	5	6·5 (0–9)	12 (9–15)	9 (2–25)
Breast	4	1 (0–3)	5·5 (3–11)	5·5 (4–11)
Wound healing	4	1·5 (0–2)	2 (1–8)	1·5 (1–17)
HPB	3	2 (0–8)	2 (0–4)	4 (1–8)
Emergency surgery	2	5·5 (0–11)	12*	6 (0–12)
Vascular	1	4*	n.a.	6*
*P* †		0·813	0·253	0·800
**Laboratory *versus* clinical research**				
Laboratory	21	2 (0–11)	6·5 (0–19)	6 (0–30)
Clinical	14	4 (0–9)	6 (3–21)	6·5 (0–25)
*P* †		0·441	0·535	0·960

Values are median (range);

* value based on one trainee. UGI, upper gastrointestinal; HPB, hepatopancreatobiliary; n.a., not applicable.

† Kruskal–Wallis test.

### Binary logistic regression

*Table* *4* shows the results of the multivariable analysis of factors independently associated with achieving three publications, both overall and by the end of ST4. No factors were independently associated with achieving three publications by the end of ST6.

**Table 4:** Multivariable binary logistic regression analysis of factors independently associated with achieving three publications

Three publications	Odds ratio	*P*
**Overall**		
Training grade	1·89 (1·01, 3·52)	0·045
OOPR	6·55 (2·04, 21·04)	0·002
**End of ST4**		
OOPR before HST	9·33 (1·80, 48·38)	0·008

Values in parentheses are 95 per cent confidence intervals. OOPR, out of programme for research; ST4, speciality training year 4; HST, higher surgical training.

## Discussion

The principal findings of this study were that general surgery HSTs had achieved a median of 3 (range 0–30) publications by their final 2 years of training. OOPR experience resulted in a sixfold greater number of peer‐reviewed publications, and boosted bibliometric profiles, with threefold stronger h‐indices and fourfold higher ResearchGate scores, in comparison with HSTs with no OOPR experience. Approximately two in five HSTs achieved set academic competencies by the end of their ST4 year, and four in five by the end of ST6. However, the observation that 20 per cent of HSTs (6 of 30) had not achieved these targets by this critical juncture is worrying, and focused countermeasures are clearly needed.

Any rational observer would likely agree that writing flair does not necessarily translate to better clinical and surgical skill. Moreover, other credentials, such as the award of a higher degree, which had been essential until the advent of the UK's Calman reforms, were deemed essential for career advancement^9^. In 2013, academic accomplishments were embedded in the JCST curriculum in general surgery, which mandated that all trainees deliver three learned society communications and publish three peer‐reviewed scientific articles in order to qualify for a CCT^2^. Thomas and colleagues^10^ reported in *BMJ Careers* in 2015 that these academic outputs were met by successful general surgery CCT applicants (2012–2013) in 88 and 94 per cent of applicants respectively. One in five trainees achieved a Master's degree, and one in two a doctoral degree. More recently, Brown and co‐workers^9^ reported a single UK Deanery's experience of general surgical trainees' academic productivity. Additional postgraduate academic qualifications were pursued by over 60 per cent of HSTs, and the added value associated with doctoral higher degrees was evidenced by distinctly stronger academic profiles; scientific publication numbers were eightfold higher, first author publications twofold higher, and citations 15‐fold higher, translating into h‐indices that were sixfold stronger^9^.

International perspectives vary. In India, a surgical dissertation has been mandatory for over 60 years, but the most crucial part of the fellowship remains the clinical examination, mandated by the Indian Medical Council to assess clinical skills^11^. In Australia and New Zealand, the Royal Australasian College of Surgeons introduced a thesis requirement in some surgical disciplines in 2008; time out of programme is not required, but successful thesis award by examination is expected, although the final examination continues to emphasize clinical judgement^12^. USA subspecialty certification is by specialty boards, and many surgical specialties require universal research training; although optional in general surgery^13^, time accredited must subsequently result in a peer‐reviewed journal publication to qualify for board certification.

To provide balance, there are clear and present potential obstacles to pursuing a successful period of research. In 2017, Keswani *et al*.^11^ reported impediments, including perceived pressure to be clinically productive, clerical workload, funding shortages, and work–life equilibrium. Goldstein and colleagues^12^ described a roadmap of key factors for achieving success as a surgeon–scientist, which included supportive environments, committed mentors, sound finances, and social support networks. The fiscal perspective of academia should not be underestimated, both in terms of grant capture for hardware, software, staff, trial recruitment incentives, and also as rewards for academic surgeons in terms of salary and bonuses. The impact of financial support has recently been reported by LeMaire and co‐workers, who described an ‘academic relative‐value unit (aRVU) scoring system’, whereby bonuses were awarded to faculty, based on academic productivity as self‐logged in the aRVU scoring system. Implementing aRVUs has been associated with significant increases in several key departmental academic achievement metrics: presentations increased by 49 per cent, publications by 14 per cent, total research funding by 83 per cent to US$8·4 million (€7·8 million; exchange rate 5 May 2020), National Institutes of Health funding by 467 per cent to US$3·4 million (€3·1, exchange rate 5 May 2020), industry‐sponsored clinical trials by 188 per cent, academic society committee positions by 32 per cent, and editorial leadership positions by 48 per cent^13^, ^14^, ^15^. Increasing both direct productivity and supervisory roles should, in turn, promote better mentors and attract future academic surgeons.

The nature of this study has inherent hypothetical limitations, which have been alluded to previously^9^. In particular, whether data relating to a single country are translatable and applicable on an international scale is open to conjecture. Moreover, the accuracy of ISCP‐derived data was proportionately dependent on the reliability with which surgical trainees had populated their online portfolios. Similarly, internet‐based search engines such as PubMed do not acknowledge or capture research outputs universally. In contrast, the study's strengths are its originality, consisting of a consecutive 10‐year cohort of UK HSTs, the results of which carry statistical power.

Academic reach has long been universally fundamental to career advancement in medical arenas, and the metrics to judge such performance commonly default to peer‐reviewed publication quantity and quality, yet the peer‐review process is widely recognized as profoundly flawed^9^. Formal OOPR experience is advantageous in many ways, but academic units of surgery within contemporary British university structures are on the wane, arguably because of the UK Research Excellence Framework funding process^16^. Future surgical leaders, regardless of demographics, should have access to clinical research practice integrated within professional training, not necessarily to obtain additional credentials, but because of the need to consider, reflect, and act upon new and emerging evidence that may inform and change clinical practice. Surgical curricula are under continual review, and the value of higher degrees as a postgraduate currency will likely fall. If CCT competencies are to include academic profiles, training programmes should incorporate research training within workforce planning. The advantage to international healthcare services is the development of multidisciplinary, adept clinicians, able to encourage translational science and stimulate a healthcare system capable of existential flexibility.
